# Paraoxonase activity in metabolic syndrome in children and adolescents

**DOI:** 10.22088/cjim.9.2.116

**Published:** 2018

**Authors:** Arati Adhe-Rojekar, Mukund Ramchandra Mogarekar, Mohit Vijay Rojekar

**Affiliations:** 1 Department of IVF, PD Hinduja National Hospital and Research Center, Mahim Mumbai. India; 2 Department of Biochemistry, SRTR Govt Medical College, Ambajogai. India.; 3 Department of Biochemistry, Rajiv Gandhi Medical College, Thane. India

**Keywords:** Paraoxonase1, Arylesterase, Lactonase, Area under Curve, ROC curve, Regression Analysis

## Abstract

**Background:**

Metabolic syndrome (MetS) is a collection of various interrelated risk factors that appear to have an impact as development of atherosclerotic cardiovascular disease (CVDs). Epidemic of childhood and adolescent’s obesity has increased interest in the metabolic syndrome (MS) due to the potential projection into adulthood. The prevalence of MS in adolescents has been estimated to be 6.7% in young adults and 4.2% in adolescents. We aimed to study the MetS in children and adolescents with respect to metabolic changes.

**Methods:**

The international Diabetes Federation criteria were used for the selection of cases. Serum paraoxonase 1 (PON1) activities were measured using spectrophotometer. Statistical analysis was done using MyStat statistical software.

**Results:**

Serum PON1 arylesterase (ARE) and lactonase (LACT) activities were found to be reduced significantly in patients with MetS than in controls. Regression analysis showed a significant correlation between PON1 activities and body mass index. Area under curve (AUC) found to increase from HDL to PON1 ARE to PON1 LACT.

**Conclusions:**

From the present study, it is clear that in children and adolescents, reduction in PON1 activities in MetS is mainly due either to abnormalities with synthesis or secretion of HDL cholesterol or oxidative stress as a consequence of excess production of the free radicals. This study also iterates that it is the quality and not the quantity of HDL cholesterol which is important while studying the pathophysiology of MetS.

The lifestyle changes are leading to dreadful metabolic disorders. The metabolic syndrome is one of them. The metabolic syndrome (MetS) is a collection of various interrelated risk factors that appear to have impact as the development of atherosclerotic cardiovascular disease (CVDs) (1). It is linked with marked increase in cardiovascular risk associated with clustering of risk factors. The central features of the metabolic syndrome are insulin resistance, visceral adiposity, atherogenic dyslipidemia and endothelial dysfunction (2, 3). 

 It is more disastrous in children and adolescents. Epidemic of childhood and adolescent obesity has increased the interest in the metabolic syndrome (MS) due to the potential projection into adulthood. The prevalence of the MS in adolescents has been estimated to be 6.7% in young adults and 4.2% in adolescents. The definitions of metabolic syndrome include those proposed by the World Health Organization (WHO), the European Group for the Study of Insulin Resistance (EGIR), the US National Cholesterol Education Program (ATPIII) and International Diabetes Federation (IDF) (4-7). Along with obesity, various other factors increase susceptibility for MetS (8). All individual components of MetS are risk factors for CVD.

**PATHOPHYSIOLOGY OF METABOLIC SYNDROME**

**Insulin resistance and Obesity: **Insulin resistance (IR) and obesity seem to be important risk factors for MetS. IR is contributed by free fatty acids (FFAs) which reduce insulin sensitivity in muscle by inhibiting insulin-mediated glucose uptake. In the liver, FFAs increase the production of glucose, triglycerides (TG) and very low density lipoproteins (VLDL). Consequently, there is reduced glycogen formation, increased lipid accumulation in TG, lipolysis and increased inflammatory cytokines (9-12). Obesity is associated with the development of oxidative stress & IR (13-15).

**PARAOXONASE: **Paraoxonase (PON) is a family of Ca^++^ dependent enzymes namely PON1, PON2 and PON3 the gene which is located on chromosome 7q21.3-22.1 (16, 17). Paraoxonase1 (PON1) is exclusively associated with HDLc and is a genetically polymorphic enzyme. It plays a vital role in the prevention of microvascular complications due to oxidative stress and against various toxic chemicals (18). The HDL oxidation is stopped by PON1-mediated hydrolysis of lipid peroxides (19). Thus, the aim of the present study was to estimate PON1 arylesterase (ARE), lactonase (LACT) activity in patients with metabolic syndrome and to compare PON1 activities in children and adolescents with metabolic syndrome and the controls.

## Methods

The study was approved by the Institutional Ethics Committee and conducted during December 2014 to May 2015 in tertiary care hospital affiliated to a rural medical college in Maharashtra, India. A total of 124 subjects were enrolled in the study. Sample size was calculated using 2-sided 95% confidence interval and 80% power. Using the mean and SD form of our pilot study, the necessary sample size came to be 56. Therefore a total of 62 subjects were enrolled as cases and controls. Cases were selected randomly to avoid the selection bias from the patients visiting the hospital. For randomization, random number generator software was used. Cases were included as per criteria put forth by IDF (7). Subjects were eligible if they were healthy, age between 4 and 20 years, with a body-mass index (BMI, the weight in kilograms divided by the square of the height in meters) that exceeded the 97th percentile for their age and sex. Exclusion criteria were the known presence of diabetes and the use of medication that alters blood pressure or glucose or lipid metabolism. As per the Helsinki declaration, written informed consent was taken from parents or legal guardians of all the participants. With all aseptic precautions early morning fasting blood samples were collected by venipuncture from the cases. 

From the control subjects, blood samples were collected at the time of their routine clinical visits through venipuncture. Samples were analyzed immediately after processing. Serum PON1 ARE activity was measured spectrophotometrically at 270 nm with 3 mL buffer-substrate solution containing 20 mmol Tris-hydroxymethyl (HCI) buffer and 4 mmol phenylacetate as substrate at pH 8.0 and 5 μl serum. Serum PON1 LACT was measured spectrophotometrically at 270 nm with 2 mL buffer substrate solution containing 50 mmol Tris-HCl buffer and 1 mmol dihydroxycoumarin as substrate at pH 8.0 and 10 μl serum (20, 21). Normality of the distribution of the arylesterase and lactonase activity was assessed by Shapiro-Wilk test. Two sample t-test was applied for hypothesis testing. Results were expressed as mean±SD for all continuous variables. The statistical significance level was set at 0.05. There were no differences between the two groups with regard to age and sex. The results obtained were analyzed using Mystat statistical software.

## Results

Serum PON1 arylesterase activity reduced significantly in cases of MetS than in controls. Similarly, serum PON1 lactonase activity reduced significantly in cases of MetS. The results show that arylesterase and lactonase singly can correlate well with BMI and their combination even more so. The study parameters are shown in table 1.

**Table 1:** The study parameters

**Parameter**	**Cases** **(n=62)** **(mean±SD)**	**Controls** **(n=62)** **(mean±SD)**	**P-value**
Age (Yrs)	16.8±2.12	17.3±1.84	0.245
BMI (Kg/m^2^)	31.52±3.2	19.1±3.53	<0.001
HDL Cholesterol (mg/dl)	32.17±3.24	34.37±4.16	0.0025
PON1 Arylesterase (kU/L)	97.36±23.09	115.63±24.43	0.0003
PON1 Lactonase (U/L)	5.58±1.34	6.69±1.63	0.0005

BMI: Body mass index, PON: Paraoxonase, SD: Standard deviation, HDL: High-density lipoprotein

**Figure 1:**
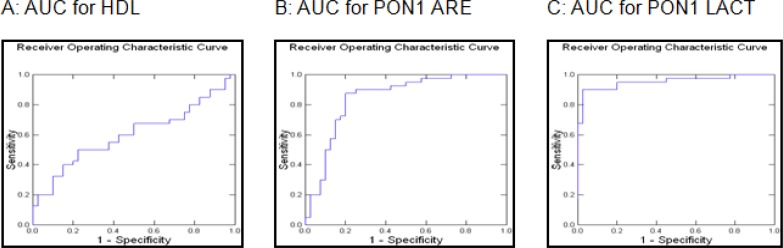
Receiver operator characteristic (ROC) curve for HDL, ARE & LACT

The receiver operator characteristic (ROC) curve shows an increase in area under curve (AUC) as we go from HDL to PON1 ARE to PON1 LACT. Cox & Snell R^2 ^as well as Naglekerke’s R^2 ^also increased in the similar manner. It is shown in figure 1 (A, B, C). From the ROC curve, it is clear that PON1 activities are better predictors of metabolic syndrome in children and adolescents than lipid profile or HDL levels. Logistic regression analysis shows area under curve (AUC) as 0.623, 0.851 and 0.953 for HDL, PON1 ARE and PON1 LACT, respectively. Cox & Snell R^2 ^comes out to be 0.056, 0.336 and 0.553 respectively for HDL, PON1 ARE and PON1 LACT. Nagelkerke’s R^2 ^is 0.075, 0.448 and 0.954 for HDL, PON1 ARE and PON1 LACT respectively.

**AUC**: area under curve, **HDL**: high density lipoprotein, **PON1**: paraoxonase 1, **ARE**: arylesterase, **LACT**: lactonase

Linear regression analysis shows R=-0.495 indicating significant correlation between arylesterase and BMI (p<0.001). Similarly, serum lactonase activity of PON1 also reduced in BMI. Its linear regression analysis showed R=-0.646 suggesting significant correlation between lactonase and birth weight (p<0.001). Both these show the significant negative correlation with BMI.

## Discussion

To the best of our knowledge, this is the first study considering the correlation of metabolic syndrome with serum arylesterase and lactonase activities in combination in children and adolescent population. Plasma PON1 activity was found to be altered, usually decreased in number of pathological conditions and diseases (22). In the present study, we found that there is a significant reduction in the PON1 arylesterase and lactonase activity in children and adolescents with metabolic syndrome.

It appears that the arylesterase and lactonase activities of PON1 are new and independent markers for MetS. Some of the researchers found the significant negative correlation between oxidative stress and PON1 levels (23). It can be hypothesized that the oxidative damage to the lipoproteins especially HDL leads to reduced PON1 activity. Biochemical findings of metabolic syndrome show the reduced HDL cholesterol. Decreased concentration of HDLc is correlated with reduced PON1 activities (24). Our results depict though not very significant the positive correlation of HDLc with arylesterase and lactonase.

The reduced PON1 activities are supposed to be the consequence of dysfunctional or modified HDLc which is responsible for the reduced antioxidant capacity (25). As PON1 prevents oxidation of LDL and HDL, qualitative changes in lipoproteins coupled with reduced HDLc concentration leading to reduced PON1 activity, render them more susceptible to oxidative damage. Progressive decline in enzymatic capacity of PON1 with disease severity of MetS inactivates PON1 (26) as a likely consequence of oxidative stress in the metabolic syndrome, exceeding the antioxidant capacity of the enzyme can be the possible explanation of reduced PON1 activity. Literature states severity of MetS is directly proportional to oxidative stress which inactivates PON1 function (26). But at the same time, the possibility that low PON1 function fails to provide an efficient protection against MetS relates oxidative damage that cannot be excluded. PON1 is capable of protecting lipoproteins against the effect lipid peroxidation by degrading specific oxidized cholesteryl esters and phospholipids, and antioxidant properties of HDL have been attributed, at least partially to PON1 (27). This may be the possible explanation for reduced PON1 activity found in MetS. Some researchers revealed that a low PON1 concentration is typical in MetS and may significantly lower HDLc concentration. It has also been proposed that these results be taken into account not only the HDL quantity but also HDL quality, which could be reflected, at least in part by PON1 concentration or activity (28). This is because PON1 is HDL-associated enzyme. This supports the findings that PON1 correlates well with MetS.

The reductions in the activities of PON1 found in the present study and their relationship to HDL-cholesterol suggest that the decrease of these serum enzymes may participate in the pathogenesis of metabolic syndrome. The altered PON1 arylesterase and lactonase activities in metabolic syndrome could have two possible explanations. One is that serum PON1 activities may be lowered as a result of an altered synthesis or secretion of HDL-cholesterol. The other one is the overproduction of the free radicals. Free radicals are disproportionately formed in metabolic abnormalities by oxidation and subsequent oxidative degradation of proteins. Insulin resistance seems to stimulate endothelial superoxide anion production via nicotinamide adenine dinucleotide phosphate hydrolase (29) and hence can worsen the degree of oxidative stress. Taken together, these observations strongly suggest that the activation of oxidative-stress pathways is a key component of the metabolic syndrome. In family history of metabolic syndrome, obesity is not taken into consideration in the present study which may be a possible limitation of the study. But the probability of affecting the result of the present study is very small.

In conclusion from the present study, it is clear that the reduction in PON1 activities in MetS mainly due either to abnormalities with synthesis or secretion of HDLc or oxidative stress which is a consequence of excess production of the free radicals. This study also iterates that it is the ‘quality’ and not the quantity of HDLc which needs to be taken into account while studying the pathophysiology of MetS and PON1 effectively put forth the ‘quality ’ of HDLc. Therefore our study recommends PON1 as a tool to assess the severity of MetS.
